# Time for a Change: From Exposure Assessment to Exposure Science

**DOI:** 10.1289/ehp.11595

**Published:** 2008-07

**Authors:** Paul J. Lioy

**Affiliations:** Exposure Science Division, Environmental and Occupational Health Sciences Institute, Robert Wood Johnson Medical School–University of Medicine & Dentistry of New Jersey and Rutgers University, Piscataway, New Jersey, E-mail: plioy@eohsi.rutgers.edu

During the late 1970s the emerging field of risk assessment spawned a new area of data analysis called “exposure assessment.” It was developed to characterize a process for identifying an exposed population against a measured or estimated outcome—health risk. After publication of the 1983 “Red Book,” *Risk Assessment in the Federal Government: Managing the Process* [National Research Council (NRC) 1983], it became clear that confidence in our ability to complete complex exposure assessments was low, and “specific components in exposure assessment is complicated by the fact that current methods and approaches … appear to be medium or route specific … and very few components … could be applicable to all media” (NRC 1983). However, the Total Exposure Assessment Methodology (TEAM) study had begun (Wallace et al. 1986).

In 1987, the NRC formed the first Committee on Exposure Assessment. It was apparent during the initial meeting that committee members, whose expertise ranged from basic sciences to environmental science and to medicine, were unclear about what exposure meant. A series of discussions and a workshop eventually defined many basic scientific principles, and eventually the committee’s report, *Human Exposure Assessment for Air Pollutants* [the “White Book” (NRC 1991)], laid the foundation for further development of the field of exposure. An immediate consequence of this effort was the formation of the International Society of Exposure Analysis (ISEA), which provided a home for human exposure research and the mechanistic foundations of the field. Concurrently, a laboratory in the U.S. Environmental Protection Agency (EPA) was renamed the National Exposure Research Laboratory, the Environmental and Occupational Health Sciences Institute started the first graduate program in exposure, and faculty members were hired at some major universities to focus on human exposure research (Lioy 1999). Further, research on single-route and multiroute exposure was supported by the World Health Organization and other international organizations such as the German Environmental Survey (Hoffmann et al. 2000).

Recently, it has become apparent that many of the goals of the NRC White Book have been achieved and that analysis of exposure is becoming a priority for national and international strategies to reduce or prevent exposures. Further, the field has passed the point of solely making measurements in support of risk assessment. It has developed into a mature discipline of science, through which a theoretical framework was constructed for developing sophisticated mathematical models linking environmental science to toxicology and public health. An experimental (including observational field studies) foundation has been established to systematically examine how individuals and populations contact contaminants because of their personal activities and behaviors, the microenvironments contacted each day, and the general environment (Lioy 1990, 1999; Ott 1993). An example was the U.S. EPA’s successful National Human Exposure Assessment Survey (Sexton et al. 1995), which was used to focus exposure issues in the National Children’s Study (Needham et al. 2005). In the United States, the efforts to systematically examine problems were significantly enhanced by the U.S. EPA’s shift toward examining cumulative exposures for multiple pollutants and aggregate exposures from multiple media for a single pollutant, and the Centers for Disease Control and Prevention’s implementation of an extensive personal exposure biomonitoring program. Each methodology has reduced uncertainties outlined in the Red Book. In parallel we have seen source-to-dose modeling develop from fundamental scientific principles, such as the Modeling ENvironment for TOtal Risk [MENTOR] (Georgopoulos and Lioy 2006).

In 2006, Dana Barr, editor-in-chief of the *Journal of Exposure Science and Environmental Epidemiology*, published that journal’s working definition for the field of exposure science: “the study of human contact with chemical, physical, or biological agents occurring in their environments, and [advancement of] knowledge of the mechanisms and dynamics of events either causing or preventing adverse health outcomes” (Barr 2006). Finally, in 2008, ISEA was renamed the International Society of Exposure Science (Weisel CW, unpublished information).

In light of all the moves forward in the field and the parallel evolution of the Exposure Biology Program at the National Institute of Environmental Health Sciences (Weis et al. 2005), *Environmental Health Perspectives* will be renaming its classification for publication of research on human contact with contaminants from “exposure assessment” to “exposure science.” The change should continue to encourage and enhance the development of the new discipline and provide new avenues for publication of innovative research that directly impact the environmental health sciences, risk assessment, and risk management.

The field has passed the point of solely making measurements in support of risk assessment. It has developed into a mature discipline of science, through which a theoretical framework was constructed for developing sophisticated mathematical models linking environmental science to toxicology and public health.

## Figures and Tables

**Figure:**
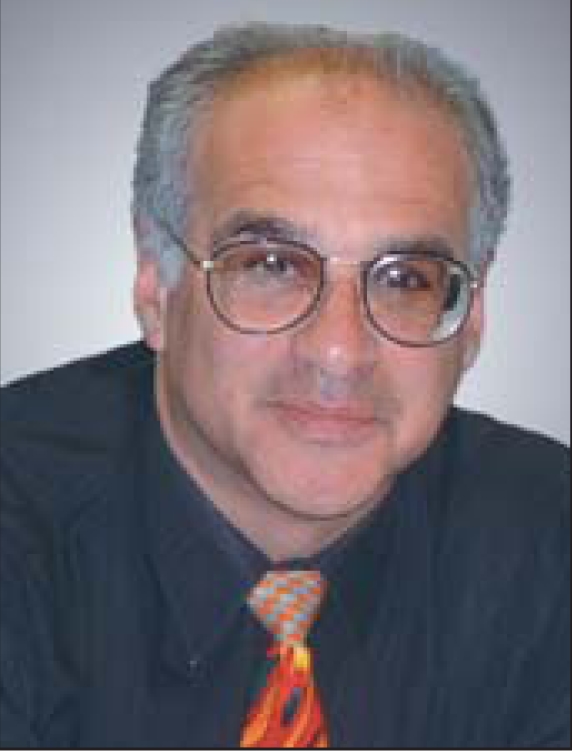
Paul J. Lioy
